# Statistical model and testing designs to increase response to selection with constrained inbreeding in genomic breeding programs for pigs affected by social genetic effects

**DOI:** 10.1186/s12711-020-00598-8

**Published:** 2021-01-04

**Authors:** Thinh Tuan Chu, Mark Henryon, Just Jensen, Birgitte Ask, Ole Fredslund Christensen

**Affiliations:** 1 Center for Quantitative Genetics and Genomics, Aarhus University, 8830 Tjele, Denmark; 2 Department of Animal Breeding and Genetics, Faculty of Animal Science, Vietnam National University of Agriculture, Hanoi, Vietnam; 3 Danish Pig Research Centre, SEGES, Axeltorv 3, 1609 Copenhagen V, Denmark; 4 School of Agriculture and Environment, University of Western Australia, 35 Stirling Highway, Crawley, WA 6009 Australia

## Abstract

**Background:**

Social genetic effects (SGE) are the effects of the genotype of one animal on the phenotypes of other animals within a social group. Because SGE contribute to variation in economically important traits for pigs, the inclusion of SGE in statistical models could increase responses to selection (RS) in breeding programs. In such models, increasing the relatedness of members within groups further increases RS when using pedigree-based relationships; however, this has not been demonstrated with genomic-based relationships or with a constraint on inbreeding. In this study, we compared the use of statistical models with and without SGE and compared groups composed at random versus groups composed of families in genomic selection breeding programs with a constraint on the rate of inbreeding.

**Results:**

When SGE were of a moderate magnitude, inclusion of SGE in the statistical model substantially increased RS when SGE were considered for selection. However, when SGE were included in the model but not considered for selection, the increase in RS and in accuracy of predicted direct genetic effects (DGE) depended on the correlation between SGE and DGE. When SGE were of a low magnitude, inclusion of SGE in the model did not increase RS, probably because of the poor separation of effects and convergence issues of the algorithms. Compared to a random group composition design, groups composed of families led to higher RS. The difference in RS between the two group compositions was slightly reduced when using genomic-based compared to pedigree-based relationships.

**Conclusions:**

The use of a statistical model that includes SGE can substantially improve response to selection at a fixed rate of inbreeding, because it allows the heritable variation from SGE to be accounted for and capitalized on. Compared to having random groups, family groups result in greater response to selection in the presence of SGE but the advantage of using family groups decreases when genomic-based relationships are used.

## Background

In typical production systems, pigs are housed in groups. Social interactions between individuals within a group influence the phenotype of each pig [1–3], such that the phenotype of an individual depends on the genotype of the animal itself (termed as the direct genetic effect, DGE) and the genotypes of the other animals in the same group (termed as the social or indirect genetic effects, SGE). SGE have been shown to account for 10 to 75% of the heritable variation of economically important traits in pigs, such as growth traits and feed intake [2, 4–7]. Thus, statistical models that include SGE might realize more responses to selection (RS) by exploiting the heritable variation from SGE and increasing the accuracy of predicted DGE.

From a production perspective, the performance of animals is important, regardless of whether the performance is due to DGE, SGE, or other effects. Because SGE are heritable and available for selection, they should be given the same weighting as DGE in the breeding goal. For instance, the total breeding value (TBV) of an individual in a group of $n$ animals is: $u_{TBV} = 1*u_{D} + \left( {n - 1} \right)*u_{S}$, where $u_{TBV}$, $u_{D}$ and $u_{S}$ are the TBV, DGE, and SGE of the individual, respectively [8]. Theoretical and empirical studies have demonstrated that a selection program that takes SGE into account increases RS [8–10]. However, these studies did not consider rate of inbreeding. Selection accounting for SGE leads to higher rates of inbreeding [11], which could generate rapid and higher short-term RS, but not necessarily long-term RS. Therefore, the use of SGE in selection programs should be investigated under a restricted rate of inbreeding, for example, using optimum-contribution selection (OCS) [12–14]. In addition, ignoring SGE when they are present can reduce the accuracy of the predicted DGE because the statistical model is mis-specified. However, in empirical studies, the use of correctly specified models that include SGE for selection has shown inconsistent results on predictive ability of DGE [4, 5, 15] and RS [1, 16]. These inconsistent results might be related to how the predictive ability of the models was investigated, i.e., based on the correlation between predicted DGE and phenotypes corrected for fixed effects. In empirical data, true breeding values and true variance components are not known. Investigating properties of models by stochastic simulation overcomes this limitation. Here in the case of breeding programs for pigs, we hypothesized that inclusion of SGE in the prediction model would improve RS at a fixed rate of inbreeding by using the correctly specified model and exploiting SGE for selection.

Allocating related animals to groups when using a model that includes SGE increases RS [10], decreases standard errors on variance estimates [17], and increases the accuracy of predicted genetic effects [18] for traits that are affected by social interactions. The increased RS obtained by increasing the number of related animals in the groups is mainly attributed to an increase in the accuracy of the predicted SGE [10, 19]. A review by Ellen et al*.* [19] demonstrated that RS is maximized when animals allocated to groups come from the same family but in that case, the genetic parameters for DGE, SGE, litter effects, and pen effects are not identifiable. To strike a balance between relatedness and effect identifiability, Ødegård and Olesen [18] suggested a design, where each group was composed of three full-sib families, and each full-sib family contributed offspring to three groups.

Previous studies [10, 17, 18] on the effects of group composition in models with SGE used pedigree information. With pedigree-based relationships, information about the SGE of a group of unrelated animals would originate from their parents and relatives in other groups. However, information from the relatives in other groups would be less informative for predicting SGE than information from relatives that are in the same group. This explains the higher accuracy of predicted SGE based on family groups compared to random groups when pedigree-based relationships are used. Compared to pedigree-based relationships, genomic-based relationships better use information from relatives, and also exploit information from animals that appear to be unrelated based on pedigree [20] to predict SGE. This may remove the advantage of having family groups compared to random groups. Thus, we hypothesized that RS is expected to be similar for random and family groups when genomic-based relationships are used.

We tested the aforementioned two hypotheses by: (1) comparing the use of statistical models with and without SGE for estimation of genetic effects and selection for traits affected by SGE in a pig breeding program, and (2) comparing groups that were randomly selected with groups that were composed of families when pedigree- versus genomic-based relationships are used in the statistical models. These comparisons were investigated for different magnitudes of SGE and correlations between SGE and DGE. In all cases, the rate of inbreeding was controlled at 1% per generation based on pedigree-based relationships.

## Methods

### Breeding program designs

The stochastic simulation program ADAM [21] was used to simulate a breeding program for pigs. The simulated genome was 30 Morgan long and consisted of 18 pairs of autosomes, 54,218 biallelic markers and 2000 quantitative trait loci (QTL). The genome was similar to that used by Henryon et al*.* [12], except that the 2000 biallelic QTL were randomly chosen from the 7702 QTL that were simulated in [12]. The breeding program was run for 10 discrete generations ($t$ = 1,…,10). At generations $t$ = 1 and 2, parents (30 sires and 600 dams) were randomly selected from the base population and the offspring of generation $t$ = 1, respectively. Each sire mated with 20 dams, and each dam mated with one sire only. Each dam produced six offspring, three males and three females. Thus, for each generation, 3600 offspring were candidates for selection. At generations $t$ = 2,…,10, offspring were allocated to groups of 12 individuals. The phenotypes of individuals were identified before selection (mimicking traits such as average daily gain or feed efficiency). Depending on the scenarios, at generation $t$ = 3, variance components were estimated using pedigree-based animal models from the dataset that had 7200 animals, with records from 1200 litters and a total of 7830 animals in the pedigree. In the other scenarios, true variance components were used. The estimated or true variance components were used to predict the genetic effects with pedigree-based or genomic-based best linear unbiased prediction (BLUP) in generations $t$ = 3,…,10. Estimated breeding values (EBV) for selection were based on predicted genetic effects, either TBV or DGE.

Selection in generations $t$ = 3,…,10 used optimum-contribution selection (OCS) procedures that controlled the rate of inbreeding at 0.01 per generation [12, 13]. To realize long-term RS, pedigree information rather than genomic information was used to compute relationships and control the rate of inbreeding, as recommended by Henryon et al*.* [12]. To save computation time, pre-selection based on EBV was carried out before optimization of the OCS procedures; specifically, 600 males and 900 females were pre-selected from 1800 male and 1800 female candidates in each generation. The pre-selection step does not reduce long-term RS [22]. The optimum number of matings, as defined by OCS, was fixed for selected dams, but not for selected sires for biological and logistical reasons [22]. Consequently, 600 dams and a number of sires determined by OCS were selected for mating. For the genomic selection scenarios, all animals at generations $t$ = 1,…,10 were genotyped, and genomic-based BLUP was used to compute EBV. Each scenario was replicated 100 times.

### Trait simulation

The phenotype of the trait for individual $i$ was simulated based on the following model:

1 $$y_{ikm} = u_{{D_{ikm} }} + \mathop \sum \limits_{j = 1, j \ne i}^{n} u_{{S_{jk} }} + c_{{p_{k} }} + c_{{l_{m} }} + e_{ikm} ,$$

where $y_{ikm}$ is the phenotype of animal $i$ in pen $k$, and from litter $m$; $u_{{D_{ikm} }}$ is the DGE of animal $i$; $\mathop \sum \nolimits_{j = 1, j \ne i}^{n} u_{{S_{jk} }}$ is the sum of the SGE of animals $j$ in pen $k$ with $n$ individuals, and animal $j$ differs from animal $i$; $c_{{p_{k} }}$ is the permanent environmental effect of pen $k$; $c_{{l_{m} }}$ is the permanent environmental effect of litter $m$; and $e_{ikm}$ is the environmental term for animal $i$. Pen and group were used as interchangeable terms in this study. Each animal had two genetic effects: (1) DGE affecting the phenotype of the animal itself ($u_{{D_{i} }}$), and (2) SGE affecting the phenotype of each pen mate ($u_{{S_{i} }}$). The genetic effects, DGE and SGE, of the trait were fully controlled by 2000 QTL. Epistasis and dominance effects of QTL were not simulated. The allelic effects of QTL were scaled to obtain an initial genetic covariance matrix of $$\left( {\begin{array}{*{20}c} 1 & {\sigma_{{u_{DS} }} } \\ {\sigma_{{u_{DS} }} } & {\sigma_{{u_{S} }}^{2} } \\ \end{array} } \right)$$ in the base population, where $\sigma_{{u_{D} }}^{2}$ = 1 is the variance of DGE, $\sigma_{{u_{S} }}^{2}$ is the variance of SGE, and $\sigma_{{u_{DS} }}$ is the covariance between the DGE and SGE. The allelic effects of QTL were kept constant across generations but the allele frequency at each QTL could change due to selection and drift. Pen, litter, and environmental effects were drawn from normal distributions of $N\left[ {0,\sigma_{{c_{p} }}^{2} = 0.25} \right]$, $N\left[ {0,\sigma_{{c_{l} }}^{2} = 0.25} \right]$ and $N\left[ {0,\sigma_{e}^{2} = 2.5} \right]$, respectively. Because we assumed a constant group size of 12 individuals, the environmental effect of social interactions was completely confounded with the pen effect and, therefore, the environmental effect of social interactions was not simulated separately. The levels of the pen and litter effects assumed in our study were moderate compared to those in previous studies [2, 4–7].

### Statistical models

The statistical models used to derive EBV were the classical model without SGE (CGM) and with SGE (SGM). The SGM model was the same as that used to simulate the trait (Model 1). In matrix notation, SGM is Model (2) and CGM is Model (3):

2 $${\mathbf{y}} = {\mathbf{Xb}} + {\mathbf{Z}}_{{\mathbf{D}}} {\mathbf{u}}_{{\mathbf{D}}} + {\mathbf{Z}}_{{\mathbf{S}}} {\mathbf{u}}_{{\mathbf{S}}} + {\mathbf{W}}_{{\mathbf{p}}} {\mathbf{c}}_{{\mathbf{p}}} + {\mathbf{W}}_{{\mathbf{l}}} {\mathbf{c}}_{{\mathbf{l}}} + {\mathbf{e}},$$

3 $${\mathbf{y}} = {\mathbf{Xb^{\prime}}} + {\mathbf{Z}}_{{\mathbf{D}}} {\mathbf{u}}_{{\mathbf{D}}}^{^{\prime}} + {\mathbf{W}}_{{\mathbf{p}}} {\mathbf{c}}_{{\mathbf{p}}}^{^{\prime}} + {\mathbf{W}}_{{\mathbf{l}}} {\mathbf{c}}_{{\mathbf{l}}}^{^{\prime}} + {\mathbf{e^{\prime}}},$$

where ${\mathbf{y}}$ is the vector of individual phenotypic records; ${\mathbf{b}}$ and ${\mathbf{b^{\prime}}}$ are the vectors of the fixed effects of generations, ${\mathbf{u}}_{{\mathbf{D}}}$ and ${\mathbf{u}}_{{\mathbf{D}}}^{^{\prime}}$ are the vectors of DGE, ${\mathbf{c}}_{{\mathbf{p}}}$ and ${\mathbf{c}}_{{\mathbf{p}}}^{^{\prime}}$ are the vectors of environmental effects of pen,${\mathbf{c}}_{{\mathbf{l}}}$ and ${\mathbf{c}}_{{\mathbf{l}}}^{^{\prime}}$ are the vectors of environmental effects of litter, ${\mathbf{e}}$ and ${\mathbf{e^{\prime}}}$ are the vectors of residuals in Models (2) and (3), respectively;${\mathbf{u}}_{{\mathbf{S}}}$ is the vector of SGE; ${\mathbf{X}}$, ${\mathbf{Z}}_{{\mathbf{D}}}$, ${\mathbf{W}}_{{\mathbf{p}}}$ and ${\mathbf{W}}_{{\mathbf{l}}}$ are incidence matrices that associate fixed, DGE, pen, and litter effects, respectively, to the phenotypic records; ${\mathbf{Z}}_{{\mathbf{S}}}$ is an incidence matrix relating phenotypic records to the SGE of group mates. Vectors ${\mathbf{c}}_{{\mathbf{p}}}$, ${\mathbf{c}}_{{\mathbf{l}}}$, and ${\mathbf{e}}$ in Model (2) were assumed to be independent and follow normal distributions: ${\mathbf{c}}_{{\mathbf{p}}} \sim N\left[ {{\mathbf{0}},{\mathbf{I}}_{{\mathbf{p}}} \sigma_{{c_{p} }}^{2} } \right]$, ${\mathbf{c}}_{{\mathbf{l}}} \sim N\left[ {{\mathbf{0}},{\mathbf{I}}_{{\mathbf{l}}} \sigma_{{c_{l} }}^{2} } \right]$, and ${\mathbf{e}}\sim N\left[ {{\mathbf{0}},{\mathbf{I}}_{{\mathbf{e}}} \sigma_{e}^{2} } \right]$, where $\sigma_{{c_{p} }}^{2}$, $\sigma_{{c_{l} }}^{2}$, and $\sigma_{e}^{2}$ are the variances of pen, litter, and residual effects, respectively; ${\mathbf{I}}_{{\mathbf{p}}}$, ${\mathbf{I}}_{{\mathbf{l}}}$, and ${\mathbf{I}}_{{\mathbf{e}}}$ are identity matrices. Vectors ${\mathbf{c}}_{{\mathbf{p}}}^{^{\prime}}$, ${\mathbf{c}}_{{\mathbf{l}}}^{^{\prime}}$, and ${\mathbf{e^{\prime}}}$ in Model (3) were similarly assumed to be independent and follow normal distributions: ${\mathbf{c}}_{{\mathbf{p}}}^{^{\prime}} \sim N\left[ {{\mathbf{0}},{\mathbf{I}}_{{\mathbf{p}}} \sigma_{{c_{p}^{^{\prime}} }}^{2} } \right]$, ${\mathbf{c}}_{{{\mathbf{l^{\prime}}}}} \sim N\left[ {{\mathbf{0}},{\mathbf{I}}_{{\mathbf{l}}} \sigma_{{c_{l}^{^{\prime}} }}^{2} } \right]$, and ${\mathbf{e^{\prime}}}\sim N\left[ {{\mathbf{0}},{\mathbf{I}}_{{\mathbf{e}}} \sigma_{{e^{\prime}}}^{2} } \right]$, where $\sigma_{{c_{p}^{^{\prime}} }}^{2}$, $\sigma_{{c_{l}^{^{\prime}} }}^{2}$, and $\sigma_{{e^{\prime}}}^{2}$ are the variances of the pen, litter, and residual effects, respectively.

Vectors ${\mathbf{u}}_{{\mathbf{D}}}$ and ${\mathbf{u}}_{{\mathbf{S}}}$ in Model (2) were assumed to be jointly normally distributed: $$\left[ {\begin{array}{*{20}c} {{\mathbf{u}}_{{\mathbf{D}}} } \\ {{\mathbf{u}}_{{\mathbf{S}}} } \\ \end{array} } \right]\sim N\left[ {{\mathbf{0}},{\mathbf{U}} \otimes \left( {\begin{array}{*{20}c} {\sigma_{{u_{D} }}^{2} } & {\sigma_{{u_{DS} }} } \\ {\sigma_{{u_{DS} }} } & {\sigma_{{u_{S} }}^{2} } \\ \end{array} } \right)} \right]$$, where $\sigma_{{u_{D} }}^{2}$, $\sigma_{{u_{S} }}^{2}$, and $\sigma_{{u_{DS} }}$ are as defined for Model (1); $\otimes$ is the Kronecker product; and ${\mathbf{U}}$ is a relationship matrix between individuals that was constructed from pedigree information in the pedigree-based BLUP models or from marker data in the genomic-based BLUP models. Vector ${\mathbf{u}}_{{{\mathbf{D^{\prime}}}}}$ in Model (3) was assumed to be normally distributed: ${\mathbf{u}}_{{\mathbf{D}}}^{^{\prime}} \sim N\left[ {{\mathbf{0}},{\mathbf{U}}\sigma_{{u_{D}^{^{\prime}} }}^{2} } \right]$, where $\sigma_{{u_{D}^{^{\prime}} }}^{2}$ is the variance of DGE. In both Models (2) and (3), these genetic effects were assumed to be independent of the other effects in the model.

Variance components were estimated at generation $t$ = 3 by the average information restricted maximum likelihood estimation method using the DMUAI procedure of the DMU package [23]. Default values from DMU were used to assess convergence of the models. For instance, the Frobenius norm of the updated vector was set at less than 10^–7^, and the maximum number of iterations to maximize the likelihood function, was set at 200 for the DMUAI procedure [23]. Variance components were estimated with the pedigree-based model. Predicted genetic effects at $t$ = 3…10 were based on either pedigree-based BLUP or genomic-based BLUP, using the DMU5 procedure of the DMU package [23], which iteratively solves the mixed model equations using the preconditioned conjugate gradient method [23]. The maximum number of iterations and convergence criteria were set at 2000 rounds and 10^–8^, respectively, for the DMU5 procedure [23]. In each generation, $t$ = 3…10, predicted genetic effects for all individuals were computed using phenotypes and genomic data or pedigree from all animals in generations 1,…,$t$. The predicted genetic effects were used to compute EBV that were used for the OCS procedures, which were carried out with the EVA software [24].

### Factors investigated

The first aim of our study was to compare the RS realized by the classical model without SGE (i.e. CGM) and with SGE (i.e. SGM). For SGM, selection criteria were based on either predicted DGE or predicted TBV. The TBV of an animal was defined as the sum of DGE and ($n - 1$) times SGE, where $n$ is the number of animals in the group [8]. The combination of different statistical models to predict genetic effects and different selection criteria resulted in three breeding schemes that used: (i) SGM with selection criteria based on TBV, (ii) SGM with selection criteria based on DGE, and (iii) CGM with selection criteria based on DGE. These three breeding schemes and using either pedigree-based or genomic-based relationship matrices, were investigated for traits with different SGE variances ($\sigma_{{u_{S} }}^{2}$) and different correlations ($r_{{u_{DS} }}$) between DGE and SGE (Table 1). Combinations of these four factors yielded 36 scenarios. Variance components for use in the statistical models for these 36 scenarios were estimated based on data available at generation $t$ = 3 in the breeding program. Animals in these scenarios were allocated to groups at random.

**Table 1:** Factors investigated

Factors investigated	Comparing the use of models CGM and SGM	Comparing group composition designs
Breeding scheme	CGM-DGE; SGM-TBV; SGM-DGE	CGM-DGE; SGM-TBV; SGM-DGE
Relationships	$\mathbf{A}$; $\mathbf{G}$	$\mathbf{A}$; $\mathbf{G}$
${\sigma }_{{u}_{S}}^{2}$	0.001; 0.01	0.01
${r}_{{u}_{DS}}$	− 0.5; 0; 0.5	0
Group composition	Random	Random; four families
Variance components used	Estimated values	Estimated values; true values

CGM is the classical model without social genetic effects (SGE); SGM is the model with SGE; CGM-DGE is the scheme using CGM with predicted direct genetic effects (DGE) as selection criteria; SGM-TBV is the scheme using SGM with predicted total breeding value (TBV) as selection criteria; SGM-DGE is the scheme using SGM with DGE as selection criteria; $\mathbf{A}$ and $\mathbf{G}$ are the pedigree- and genomic-based relationship matrix, respectively; ${\sigma }_{{u}_{S}}^{2}$ is the social additive genetic variance; ${r}_{{u}_{DS}}$ is the correlation between DGE and SGE

We assumed a variance of 1 for the DGE ($$\sigma_{{u_{D} }}^{2}$$) and of 0.001 or 0.01 for SGE ($$\sigma_{{u_{S} }}^{2}$$). For economically important pig traits, the values of $$\sigma_{{u_{S} }}^{2}$$ used represent SGE with low and moderate magnitudes compared to previous studies [2, 4–7]. For example, the ratio $${\raise0.7ex\hbox{${\sigma_{{u_{S} }}^{2} }$} \!\mathord{\left/ {\vphantom {{\sigma_{{u_{S} }}^{2} } {\sigma_{{u_{D} }}^{2} }}}\right.\kern-\nulldelimiterspace} \!\lower0.7ex\hbox{${\sigma_{{u_{D} }}^{2} }$}}$$ was on average 0.01 (ranging from 0.002 to 0.044) in previous studies [2, 4–7] and the ratio of SGE to total phenotypic variance $$\left( {\frac{{\left( {n - 1} \right)^{2} \sigma_{{u_{S} }}^{2} }}{{\sigma_{P}^{2} }}} \right)$$ was on average 0.25 (ranging from 0.10 to 0.53) [2, 4–7] (see Bergsma et al*.* [2] for the formula for the total phenotypic variance $$\sigma_{P}^{2}$$), compared to ratios of 0.03 and 0.29 for $$\sigma_{{u_{S} }}^{2}$$ equal to 0.001 and 0.01, respectively, when group members are unrelated. Three values of $$r_{{u_{DS} }}$$ were used: − 0.5, 0, and 0.5. The total heritable variation can be computed as $$\sigma_{{u_{TBV} }}^{2} = \sigma_{{u_{D} }}^{2} + 2\left( {n - 1} \right)\sigma_{{u_{DS} }} + \left( {n - 1} \right)^{2} \sigma_{{u_{S} }}^{2}$$, which was equal to 0.77, 1.12, and 1.47 with $$\sigma_{{u_{S} }}^{2} = 0.001$$ and $$r_{{u_{DS} }}$$ − 0.5, 0, and 0.5, respectively, and equal to 1.11, 2.21, and 3.31, respectively, when $$\sigma_{{u_{S} }}^{2} = 0.01$$.

The second aim of our study was to compare random grouping versus family grouping. Each group consisted of 12 individuals. With random grouping, members of a group were sampled at random with respect to family and could, therefore be sibs by chance. With family grouping, members of a group were sampled from families. In the design with four families per group, each group was composed of members from four families, and each family contributed offspring equally to two groups. When the parents were non-inbred and unrelated, the average relationship between members within groups was 0.009 with random group composition, and 0.096 in groups composed of four families. The two group composition strategies were compared in various situations: models using different relationship matrices, different breeding schemes, and different assumed variance components (Table 1). Combining these four factors yielded 20 scenarios because the true variance components were not known for the CGM model. These 20 scenarios were investigated with $\sigma_{{u_{S} }}^{2} = 0.01$ and $r_{{u_{DS} }} = 0$. Variance components used for the SGM prediction model could be either true values or estimated values. In the second case, variances were estimated using a pedigree-based model at generation $t$ = 3. In both cases, breeding values were predicted at generations $t$ = 3,…,10 of the breeding program for selection. Using estimated versus true values of variance components is a relevant factor when different group composition designs are investigated, because group composition could affect the accuracy of EBV based on the ability to correctly estimate variance components. Because the true values of variance components were not known for the CGM model, the use of the true values was not investigated when comparing the CGM and SGM models.

### Response to selection

The realised RS was evaluated for each scenario as: $RS = \left( {\overline{{y_{10} }} - \overline{{y_{3} }} } \right)/\left( {10 - 3} \right)$, where $\overline{{y_{3} }}$ and $\overline{{y_{10} }}$ are the average of the phenotypes at generations 3 and 10, respectively. Means and standard errors of RS were calculated based on 100 replicates for each scenario. The rate of true inbreeding ($\Delta F_{true}$) based on identical-by-descent alleles was calculated as described in [12]: $\Delta F_{true} = \left( {1 - \exp \left( \beta \right)} \right)*100\%$, where $\beta$ is the regression coefficient of ${\text{ln}}\left( {1 - \overline{{F_{t} }} } \right)$ on $t$, where $\overline{{F_{t} }}$ is the average coefficient of true inbreeding for animals born in generations $t$ = 3,…,10.

The relative difference in RS between the two group composition strategies was compared for pedigree-based relationships *versus* genomic-based relationships using a statistical bootstrapping approach. The comparison was assessed by a value termed $$\Delta D_{A\_G} = {\raise0.7ex\hbox{${RS_{Fam}^{A} }$} \!\mathord{\left/ {\vphantom {{RS_{Fam}^{A} } {RS_{Ran}^{A} }}}\right.\kern-\nulldelimiterspace} \!\lower0.7ex\hbox{${RS_{Ran}^{A} }$}} - {\raise0.7ex\hbox{${RS_{Fam}^{G} }$} \!\mathord{\left/ {\vphantom {{RS_{Fam}^{G} } {RS_{Ran}^{G} }}}\right.\kern-\nulldelimiterspace} \!\lower0.7ex\hbox{${RS_{Ran}^{G} }$}}$$, where $$\left( {{\raise0.7ex\hbox{${RS_{Fam}^{i} }$} \!\mathord{\left/ {\vphantom {{RS_{Fam}^{i} } {RS_{Ran}^{i} }}}\right.\kern-\nulldelimiterspace} \!\lower0.7ex\hbox{${RS_{Ran}^{i} }$}}} \right)$$ is the relative difference in RS when using family grouping ($$RS_{Fam}^{i}$$) *versus* random grouping ($$RS_{Ran}^{i}$$) with pedigree-based ($$i = A$$) versus genomic-based ($$i = G$$) relationships. $$RS_{Fam}^{A}$$ was the mean of 100 samples that were obtained by sampling with replacement from 100 replicates with random grouping and the use of the pedigree-based relationships. Similarly, $$RS_{Ran}^{A}$$, $$RS_{Fam}^{G}$$ and $$RS_{Ran}^{G}$$ were the means of their corresponding scenarios. $$\Delta D_{A\_G}$$ was computed based on the sampled means. This process was repeated 5000 times. If $$\Delta D_{A\_G}$$ were greater than zero, the relative difference in RS between the two grouping strategies would be lower when using genomic- than when using pedigree-based relationships.

### Accuracy and bias of the predicted genetic effects

Accuracy and bias of the different predicted genetic effects were assessed for the animals in generation $t$ = 4. When comparing the difference in accuracy between scenarios, we found that the results for generation $t$ = 4 were similar to those for generation $t$ = 5…10; thus, only the results for generation 4 are presented. Accuracy of DGE (referring to the accuracy of predicted DGE), was the correlation between ${\mathbf{u}}_{{\mathbf{D}}}$ and $\widehat{{{\mathbf{u}}_{{\mathbf{D}}} }}$ in SGM or the correlation between ${\mathbf{u}}_{{\mathbf{D}}}$ and $\widehat{{{\mathbf{u}}_{{\mathbf{D}}}^{^{\prime}} }}$ in CGM, where vectors with a hat are predicted effects using a specific model and vectors without a hat are true effects from the simulation. Accuracy of SGE (referring to the accuracy of predicted SGE) was the correlation between ${\mathbf{u}}_{{\mathbf{S}}}$ and $\widehat{{{\mathbf{u}}_{{\mathbf{S}}} }}$ in the breeding scheme using TBV as the selection criteria from SGM. Accuracy of SGE was not reported for the scheme that used DGE from SGM as the selection criterion, because SGE was not accounted for in selection. The accuracy of the predicted genetic effects of selection criteria (GESC), which was directly related to RS, was calculated as the correlation between $\widehat{{{\mathbf{u}}_{{\mathbf{D}}} }}$ and ${\mathbf{u}}_{{{\mathbf{TBV}}}}$ for the breeding scheme using DGE from SGM as selection criterion, the correlation between $\widehat{{{\mathbf{u}}_{{{\mathbf{TBV}}}} }}$ and ${\mathbf{u}}_{{{\mathbf{TBV}}}}$ for the scheme using TBV from SGM, and the correlation between $\widehat{{{\mathbf{u}}_{{{\mathbf{D^{\prime}}}}} }}$ and ${\mathbf{u}}_{{{\mathbf{TBV}}}}$ for the scheme using DGE from CGM. Bias was computed as the regression coefficient of true on predicted values for DGE, SGE, and GESC.

### Estimates of variance components

Estimates of variance components obtained using the SGM and CGM models in generation $t$ = 3 were used to investigate how the variance of SGE was allocated to other variance components of the CGM when the model was mis-specified. For all scenarios, estimation of variance components was based on pedigree-based REML and datasets with random selection of parents. All replicates with the same model and the same group composition design were used to analyze the estimated variance components. For example, in the situation with a $\sigma_{{u_{S} }}^{2}$ of 0.01 and $r_{{u_{DS} }}$ of − 0.5, there were 400 replicates across scenarios that used the SGM and random group composition, compared to 200 replicates across scenarios that used the CGM and random group composition. A t-test was used to identify whether the estimates were statistically different from true values used for simulation. t-tests were also performed for comparisons between estimates of variance components from the SGM and CGM.

## Results

### Social genetic effects

**Fig. 1:**
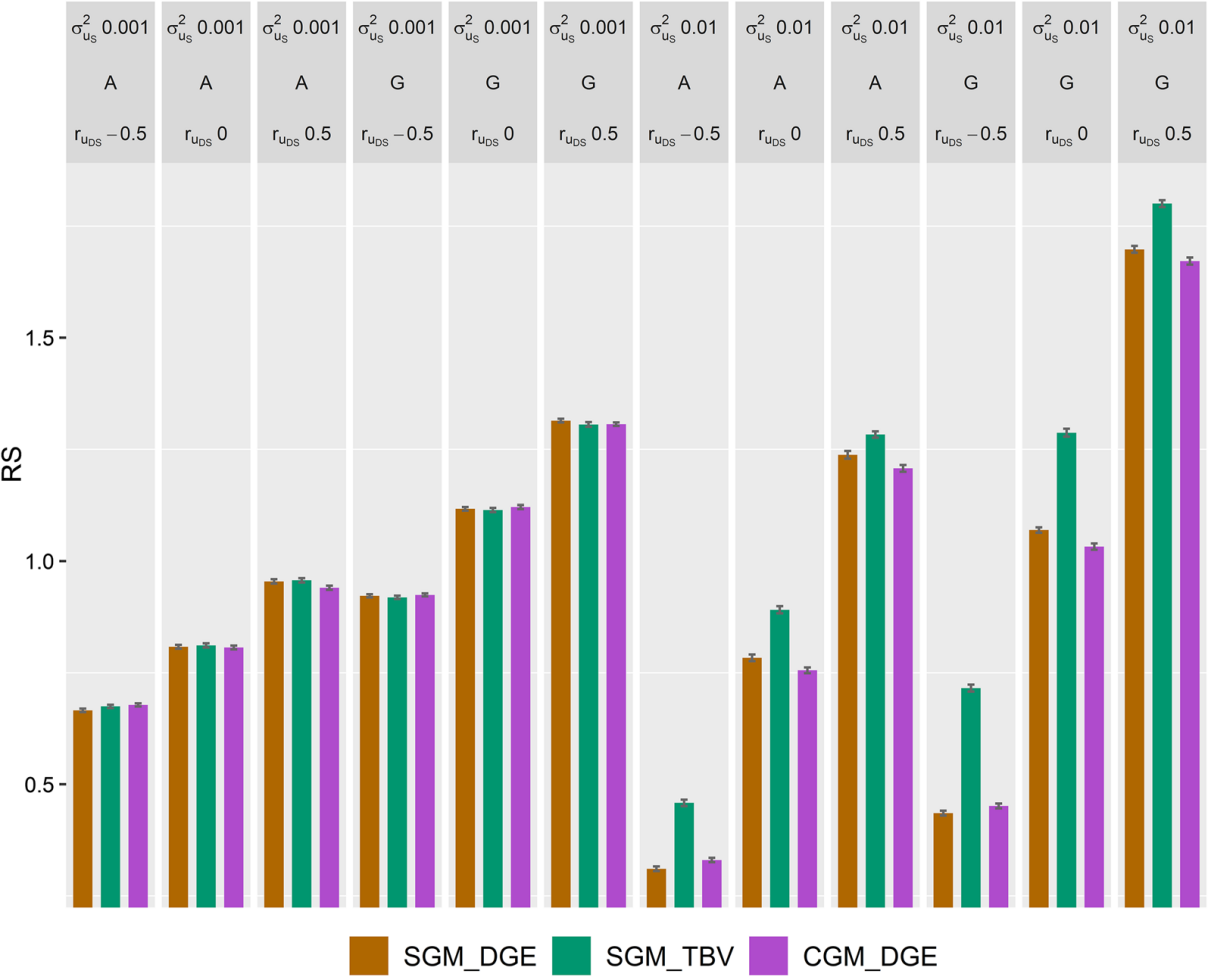
Response to selection (mean and standard error over 100 replicates) for three breeding schemes. SGM_DGE used a social genetic model (SGM) with selection criteria based on direct genetic values (DGE); SGM_TBV used a SGM with selection criteria based on total breeding values (TBV); and CGM_DGE used the classical genetic model (CGM) with selection criteria based on DGE. Breeding schemes used either pedigree- (${\mathbf{A}}$) or genomic-based (${\mathbf{G}}$) relationships to predict genetic effects. The three breeding schemes were compared for traits with SGE variance ($\sigma_{{u_{S} }}^{2}$) equal to 0.01 and 0.001 and a correlation ($r_{{u_{DS} }}$) between SGE and DGE of -0.5, 0, and 0.5. Group members were allocated at random

To compare the use of the statistical models SGM *versus* CGM, with the latter being a mis-specified model compared to the underlying simulation model, three breeding schemes were investigated: (i) SGM with selection criteria based on TBV, (ii) SGM with selection criteria based on DGE, and (iii) CGM with selection criteria based on DGE. RS was higher when genomic-based rather than pedigree-based relationships were used; however, the difference in RS between the three breeding schemes were similar for both relationships used (Fig. 1). With a $\sigma_{{u_{S} }}^{2}$ of 0.01, RS was highest when TBV from SGM were used as selection criteria. With a $\sigma_{{u_{S} }}^{2}$ of 0.01 and a $r_{{u_{DS} }}$ of 0 and 0.5, the second highest RS was obtained when the DGE from SGM were used as selection criteria. With a $r_{{u_{DS} }}$ of − 0.5, the selection criteria based on DGE using CGM had the second highest RS. When $\sigma_{{u_{S} }}^{2}$ was 0.001, RS hardly differed between the three selection strategies.

Table 2 shows the accuracies of the predicted genetic effects and the bias of predictions when $\sigma_{{u_{S} }}^{2}$ = 0.01 and $r_{{u_{DS} }}$ =  − 0.5, 0, or 0.5. Standard errors of the accuracies and the biases over 100 replicates for these scenarios are in Table S1 [see Additional file 1: Table S1]. Compared to pedigree-based relationships, the use of genomic-based relationships for prediction resulted in higher accuracies of predicted genetic effects, and generally lower bias. However, the patterns of the differences in accuracies between the three breeding schemes were similar for genomic- and pedigree-based relationships. Compared to the two alternative schemes, the scheme with selection criteria based on TBV from SGM led to the highest accuracy of GESC. With $r_{{u_{DS} }}$ =  − 0.5, GESC accuracy was lowest when using DGE from SGM as selection criteria. With $r_{{u_{DS} }}$ = 0 or 0.5, the accuracy of GESC was lowest when using CGM. The ranking of the three schemes based on the accuracy of DGE was similar to that based on the accuracy of GESC. For example, the scheme with selection criteria based on TBV from SGM had the highest accuracy of DGE compared to the alternative schemes. Although predictions of DGE were unbiased, predictions of TBV based on DGE were biased.

**Table 2:** Accuracies and bias of predicted genetic effects (mean over 100 replicates) for the three breeding schemes

$r_{{u_{DS} }}$	${\mathbf{A}}$			${\mathbf{G}}$		
	SGM_DGE	SGM_TBV	CGM_DGE	SGM_DGE	SGM_TBV	CGM_DGE
Accuracy of GESC						
− 0.5	0.201	0.318	0.204	0.244	0.437	0.269
0	0.347	0.430	0.341	0.449	0.556	0.436
0.5	0.481	0.504	0.457	0.614	0.656	0.596
Accuracy of DGE						
− 0.5	0.582	0.623	0.588	0.770	0.811	0.774
0	0.583	0.604	0.580	0.768	0.792	0.765
0.5	0.580	0.586	0.577	0.765	0.777	0.764
Bias of GESC						
− 0.5	0.39	1.00	0.39	0.38	0.98	0.41
0	0.92	1.01	0.94	0.94	0.99	0.93
0.5	1.54	1.05	1.50	1.52	1.00	1.51
Bias of DGE						
− 0.5	0.99	0.99	0.97	1.00	1.01	0.97
0	0.98	0.99	1.01	0.99	1.00	1.00
0.5	0.99	1.00	1.02	0.99	1.00	1.02

Accuracy of predicted genetic effects of selection criteria (GESC) was calculated as the correlation between GESC and true TBV. Bias of GESC was the regression coefficient of true values of TBV on predicted values of GESC

SGM_DGE used a social genetic model (SGM) with selection criteria based on direct genetic values (DGE); SGM_TBV used a SGM with selection criteria based on total breeding values (TBV); and CGM_DGE used the classical genetic model (CGM) with selection criteria based on DGE. Breeding schemes used either pedigree- (${\mathbf{A}}$) or genomic-based (${\mathbf{G}}$) relationships to predict genetic effects. The three breeding schemes were compared by making different assumptions for the trait simulated with SGE variance ($\sigma_{{u_{S} }}^{2}$) of 0.01 and correlation ($r_{{u_{DS} }}$) between SGE and DGE at − 0.5, 0, and 0.5. Group members were allocated at random. Accuracy and bias of predicted genetic effects were computed based on animals at generation $t$ = 4

With $\sigma_{{u_{S} }}^{2}$ = 0.001, accuracies of predicted genetic effects and biases are in Table S2 [see Additional file 1: Table S2]. The difference in the accuracies between the three breeding schemes was small relative to their standard errors. For 25.6 to 80.5% of the SGM predictions across replicates, the iterative solving methods for predicting genetic effects did not converge when $\sigma_{{u_{S} }}^{2}$ = 0.001, compared 1.3 to 29.8% of the predictions when $\sigma_{{u_{S} }}^{2}$ = 0.01 [see Additional file 1: Table S3]. The CGM predictions converged well for all replicates regardless of the value of $\sigma_{{u_{S} }}^{2}$

### Group composition

**Fig. 2:**
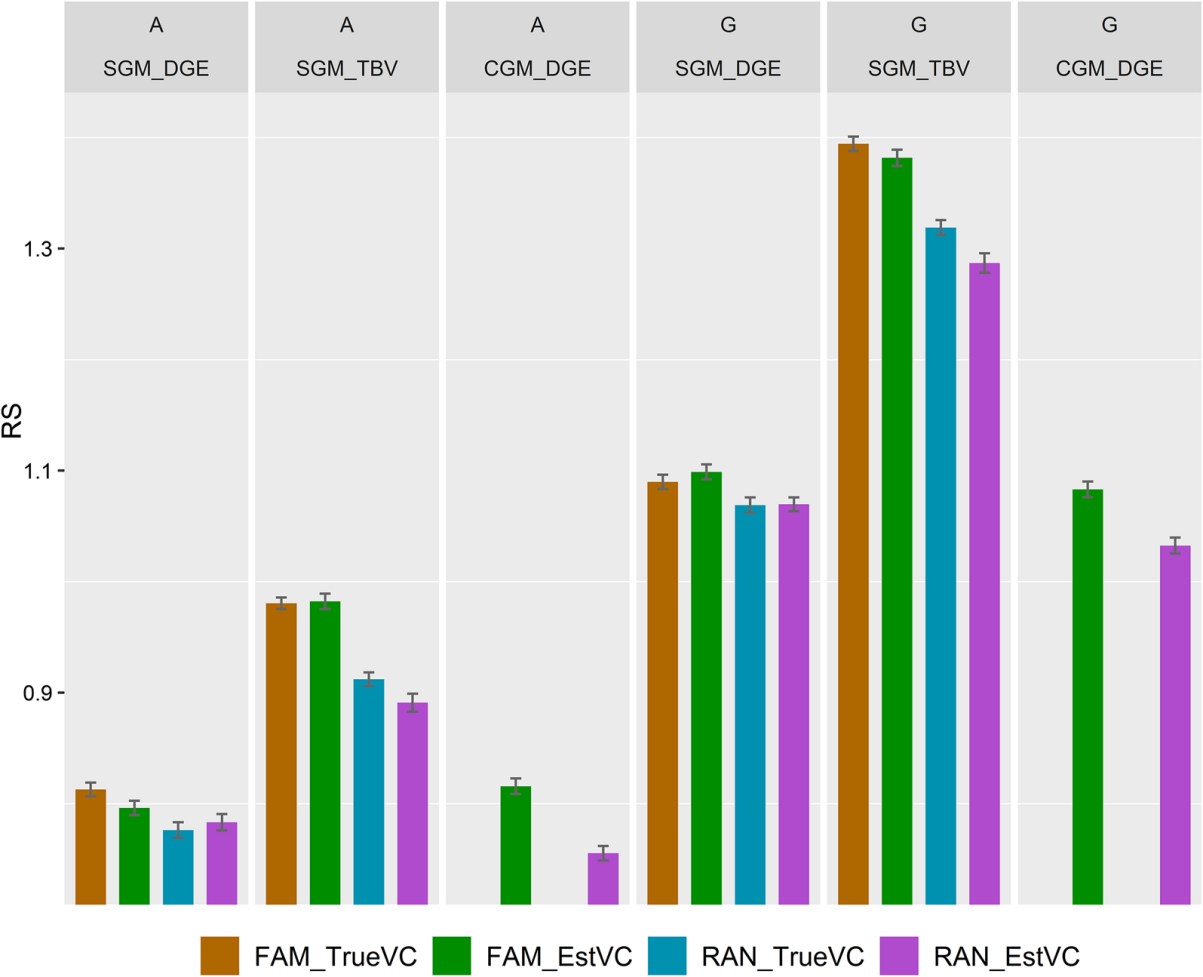
Response to selection (mean and standard error over 100 replicates) when group members were composed at random (RAN) *versus* composed of four families (FAM) per group. These two designs were compared under three breeding schemes: SGM_DGE used a social genetic model (SGM) with selection criteria based on direct genetic values (DGE); SGM_TBV used a SGM with selection criteria based on total breeding values (TBV); and CGM_DGE used the classical genetic model (CGM) with selection criteria based on DGE. Breeding schemes used either pedigree- (${\mathbf{A}}$) or genomic-based (${\mathbf{G}}$) relationships were used. The variance components used were true (TrueVC) and estimated values (EstVC). The trait was simulated with a SGE variance of 0.01 and a correlation between SGE and DGE of 0

Another aim of our study was to compare the use of groups composed at random and those composed of a few families, when using pedigree- *versus* genomic-based relationship matrices in the model for genetic evaluation. Family grouping resulted in higher RS than random grouping (Fig. 2). RS was higher for all three breeding schemes that used SGM and CGM with selection criteria based on TBV and DGE. Except when using SGM with estimated variance components and selection criteria based on DGE, the difference in RS between the two group compositions was significantly larger when pedigree-based *versus* genomic-based relationships were used (Table 3). For example, when using SGM with estimated variances and selection criteria based on TBV, the relative differences were equal to 1.10 and 1.07 for pedigree-based and genomic-based relationships, respectively. The higher relative difference obtained for pedigree-based relationships was statistically significant, with 96.6% of the $\Delta D_{A\_G}$ values being greater than zero in the bootstrapping approach. When using SGM with estimated variances and selection criteria based on DGE, 24.7% of the $\Delta D_{A\_G}$ values were greater than zero, which showed that the relative difference in RS between the two group compositions was not statistically significant between the two relationship matrices used.

**Table 3:** Relative difference in response to selection with family group composition and random group composition using pedigree-based ($\overline{{{\varvec{D}}_{{\varvec{A}}} }}$) and genomic-based ($\overline{{{\varvec{D}}_{{\varvec{G}}} }}$) relationships

Scheme	Variance components	$\overline{{D_{A} }}$	$\overline{{D_{G} }}$	Percentage of $\Delta D_{A\_G} > 0$
SGM_DGE	Estimated	1.016	1.027	24.7
SGM_DGE	True	1.047	1.020	96.7
SGM_TBV	Estimated	1.103	1.074	96.6
SGM_TBV	True	1.075	1.057	94.3
CGM_DGE	Estimated	1.080	1.049	97.1

$$\Delta D_{A\_G} = {\raise0.7ex\hbox{${RS_{Fam}^{A} }$} \!\mathord{\left/ {\vphantom {{RS_{Fam}^{A} } {RS_{Ran}^{A} }}}\right.\kern-\nulldelimiterspace} \!\lower0.7ex\hbox{${RS_{Ran}^{A} }$}} - {\raise0.7ex\hbox{${RS_{Fam}^{G} }$} \!\mathord{\left/ {\vphantom {{RS_{Fam}^{G} } {RS_{Ran}^{G} }}}\right.\kern-\nulldelimiterspace} \!\lower0.7ex\hbox{${RS_{Ran}^{G} }$}}$$, where $$RS_{Fam}^{A}$$, $$RS_{Ran}^{A}$$, $$RS_{Fam}^{G}$$, $$RS_{Ran}^{G}$$ are responses to selection (RS) obtained from the bootstrapping procedures for scenarios using group composition of families (Fam) and random (Ran) group composition for the use of pedigree- ($${\mathbf{A}}$$) and genomic-based ($${\mathbf{G}}$$) relationships. The bootstrapping procedures were repeated 5000 times, and $$\overline{{D_{A} }} = \overline{{{\raise0.7ex\hbox{${RS_{Fam}^{A} }$} \!\mathord{\left/ {\vphantom {{RS_{Fam}^{A} } {RS_{Ran}^{A} }}}\right.\kern-\nulldelimiterspace} \!\lower0.7ex\hbox{${RS_{Ran}^{A} }$}}}}$$ and $$\overline{{D_{G} }} = \overline{{{\raise0.7ex\hbox{${RS_{Fam}^{G} }$} \!\mathord{\left/ {\vphantom {{RS_{Fam}^{G} } {RS_{Ran}^{G} }}}\right.\kern-\nulldelimiterspace} \!\lower0.7ex\hbox{${RS_{Ran}^{G} }$}}}}$$ are the averages of the values from the repeated bootstrapping procedures

The comparison between the two relationships was investigated under three breeding schemes: SGM_DGE used a social genetic model (SGM) with selection criteria based on direct genetic values (DGE); SGM_TBV used a SGM with selection criteria based on total breeding values (TBV); and CGM_DGE used the classical genetic model (CGM) with selection criteria based on DGE. Variance components used were true and estimated values. The trait was simulated with an SGE variance of 0.01 and a correlation between SGE and DGE of 0

The comparison between the two group compositions was investigated using either the true or estimated variance components at generation $t$ = 3 for prediction. With random grouping, the use of true variance components for prediction resulted in higher RS than the use of estimated variance components when using selection criteria based on TBV. With family grouping, the use of true or estimated variance components resulted in only small differences in RS, because family groups resulted in more accurate estimates of variance components.

Differences in accuracy of GESC between the designs with random and family grouping were similar to the differences in RS. Specifically, with family grouping, the accuracy of GESC was higher than with random grouping (Table 4). Accuracies of GESC tended to differ more between the two group designs when pedigree-based rather than genomic-based relationships were used. With family grouping, the accuracy of SGE was higher than with random grouping. In contrast, the accuracy of DGE was lower with family grouping than with random grouping. Predictions of GESC, DGE, and SGE were generally unbiased for both group designs. Standard errors of accuracy and bias over 100 replicates of these scenarios are in Table S4 [see Additional file 1: Table S4].

**Table 4:** Accuracies and bias of predicted genetic effects (mean over 100 replicates) when group members were composed at random *versus* composed of four families per group

Scheme	${\mathbf{A}}$				${\mathbf{G}}$			
	Fam_T	Fam_E	Ran_T	Ran_E	Fam_T	Fam_E	Ran_T	Ran_E
Accuracy of GESC								
SGM_DGE	0.369	0.369	0.358	0.347	0.463	0.459	0.453	0.449
SGM_TBV	0.495	0.478	0.436	0.430	0.617	0.610	0.566	0.556
CGM_DGE	n/a	0.377	n/a	0.341	n/a	0.463	n/a	0.436
Accuracy of DGE								
SGM_DGE	0.570	0.574	0.586	0.583	0.767	0.763	0.772	0.768
SGM_TBV	0.611	0.603	0.607	0.604	0.79	0.794	0.789	0.792
CGM_DGE	n/a	0.577	n/a	0.580	n/a	0.765	n/a	0.765
Accuracy of SGE								
SGM_TBV	0.444	0.430	0.366	0.365	0.563	0.549	0.484	0.451
Bias of GESC								
SGM_DGE	1.01	0.99	0.95	0.92	0.97	0.97	0.94	0.94
SGM_TBV	1.00	1.01	0.97	1.01	0.98	1.00	0.98	0.99
CGM_DGE	n/a	1.02	n/a	0.94	n/a	0.97	n/a	0.93
Bias of DGE								
SGM_DGE	0. 99	0.99	0.99	0.98	0.99	0.99	0.99	0.99
SGM_TBV	0.99	0.98	0.99	0.99	0.98	1.00	0.99	1.00
CGM_DGE	n/a	1.01	n/a	1.01	n/a	0.98	n/a	1.00
Bias of SGE								
SGM_TBV	1.00	0.97	0.97	1.05	1.00	1.01	0.98	1.07

Fam_T and Fam_E are the scenarios using family (Fam_) group composition and true (T) or estimated (E) variance components for prediction, respectively. Ran_T and Ran_E are the scenarios using random (Ran_) group composition and T or E variance components, respectively

These two designs were compared under three breeding schemes: SGM_DGE used a social genetic model (SGM) with selection criteria based on direct genetic values (DGE); SGM_TBV used a SGM with selection criteria based on total breeding values (TBV); and CGM_DGE used the classical genetic model (CGM) with selection criteria based on DGE. Breeding schemes used either pedigree- (${\mathbf{A}}$) or genomic-based (${\mathbf{G}}$) relationships to predict genetic effects. The trait was simulated with an SGE variance of 0.01 and a correlation between SGE and DGE of 0.

Accuracy of SGE and bias of SGE are not available (n/a) for model CGM.

### Variance component estimates

Table 5 shows estimates of variance components based on SGM and CGM at generation $t$ = 3 based on the pedigree-based relationship matrix. Three scenarios used a random grouping with $r_{{u_{DS} }}$ =  − 0.5, 0, or 0.5 used for simulation, while the fourth scenario used family grouping with $r_{{u_{DS} }}$ = 0. Variance component estimates from SGM, which was the true model, were not statistically significantly different from the true variances, except for estimates of the variance of DGE with $r_{{u_{DS} }}$ =  − 0.5 and 0.5. Statistical significance of the differences between the true and estimated variances from CGM varied depending on the variance component evaluated. Estimates of variances of DGE and pen effects from CGM were statistically significantly different from their true values, while those of litter effects were not. The variances estimated from SGM and CGM were statistically significantly different for pen effects for all four scenarios (P < 0.0001). The estimates of the variance of DGE from SGM and CGM were not statistically significantly different with family grouping, but were with random grouping. Variance estimates from SGM and CGM were generally not statistically significant different for litter effects.

**Table 5:** Variance components estimated at generation ${\varvec{t}}$ = 3 using social (SGM) and classical genetic (CGM) models for group members composed at random or families

Parameter	Simulated values	Estimates^a^ from SGM	(SD)	Estimates^b^ from CGM	(SD)	SGM-CGM^c^
Random group and $r_{{u_{DS} }}$ = − 0.5						
DGE variance	1	0.975*	0.145	1.023	0.142	− 0.051***
Covariance of DGE and SGE	− 0.05	− 0.049	0.018			
SGE variance	0.01	0.010	0.004			
Litter variance	0.25	0.251	0.045	0.245	0.047	0.006
Pen variance	0.25	0.249	0.050	0.287***	0.035	− 0.037***
Residual variance	2.50	2.500	0.094	2.543***	0.086	− 0.044***
Random group and $r_{{u_{DS} }}$ = 0						
DGE variance	1	1	0.141	0.961**	0.143	0.039*
Covariance of DGE and SGE	0	− 0.001	0.021			
SGE variance	0.01	0.010	0.005			
Litter variance	0.25	0.245	0.044	0.256	0.043	− 0.011*
Pen variance	0.25	0.250	0.058	0.350***	0.038	− 0.1***
Residual variance	2.50	2.492	0.089	2.516	0.099	− 0.024*
Random group an $r_{{u_{DS} }}$ = 0.5						
DGE variance	1	0.980*	0.142	0.929***	0.137	0.050^**^
Covariance of DGE and SGE	0.05	0.048	0.021			
SGE variance	0.01	0.009	0.005			
Litter variance	0.25	0.247	0.045	0.248	0.045	0.000
Pen variance	0.25	0.252	0.064	0.412***	0.041	− 0.160***
Residual variance	2.50	2.501	0.088	2.478**	0.083	0.023*
Family group and $r_{{u_{DS} }}$ = 0						
DGE variance	1	0.987	0.154	0.989	0.154	− 0.002
Covariance of DGE and SGE	0	0	0.016			
SGE variance	0.01	0.010	0.003			
Litter variance	0.25	0.250	0.054	0.251	0.052	− 0.001
Pen variance	0.25	0.251	0.065	0.433***	0.049	− 0.181***
Residual variance	2.50	2.492	0.093	2.506	0.086	− 0.014

^a^Value was the mean (standard deviation) over 400 replicates for variance components estimated from SGM

^b^Value was the mean (standard deviation) over 200 replicates for variance components estimated from CGM. One sample t-test was carried out to compare the estimates from SGM and CGM with the simulated values

^c^Two sample t-test was carried out to compare the estimates from SGM with those from CGM

P-value indicates the significance: *** < 0.0001, ** < 0.001, and * < 0.01

The trait was simulated with an SGE variance of 0.01 and correlations of -0.5, 0 and 0.5 between SGE and DGE

## Discussion

### Social genetic effects

With $\sigma_{{u_{S} }}^{2} = 0.01$, i.e. SGE of moderate magnitude, our results showed that RS increased considerably with the use of SGM and predictions of TBV as selection criteria compared to the use of predictions of DGE only, and compared to the use of CGM. This result confirmed our hypothesis that SGM models improve RS by exploiting SGE for selection. However, inclusion of SGE in the prediction model did not consistently increase RS for different values of $r_{{u_{DS} }}$. With $\sigma_{{u_{S} }}^{2}$ = 0.001, i.e. SGE of low magnitude, inclusion of SGE in SGM did not improve RS, which might be due to convergence problems.

With $\sigma_{{u_{S} }}^{2} = 0.01$, the breeding scheme using predictions of TBV from SGM as selection criteria led to considerably higher RS compared to the alternative schemes because using SGE increases the heritable variation available for selection, accounts for the possible negative correlation between DGE and SGE in selection, and increases the accuracy of selection. The total heritable variation that was explored in the scheme using selection criteria based on TBV was: $\sigma_{{u_{TBV} }}^{2} = \sigma_{{u_{D} }}^{2} + 2\left( {n - 1} \right)\sigma_{{u_{DS} }} + \left( {n - 1} \right)^{2} \sigma_{{u_{S} }}^{2}$. In comparison, the heritable variation that was explored for selection in the scheme with selection criteria based on DGE was $\sigma_{{u_{D} }}^{2}$ when using SGM and $\sigma_{{u_{D}^{^{\prime}} }}^{2}$ when using CGM, where $\sigma_{{u_{D}^{^{\prime}} }}^{2} = \sigma_{{u_{D} }}^{2} - 2\sigma_{{u_{DS} }} + \sigma_{{u_{S} }}^{2}$ (see “Appendix” section). The derivation of $\sigma_{{u_{D}^{^{\prime}} }}^{2}$ in “Appendix” section was based on several assumptions such as random grouping and no litter variance but was in agreement with Chen et al. [5]. When SGE was not used in selection, the correlation between DGE and SGE was not exploited. Selection based on DGE only, could lead to negative RS [3, 9] if $\left( {\sigma_{{u_{D} }}^{2} + \left( {n - 1} \right)\sigma_{{u_{DS} }} } \right) < 0$ based on the formula of Bijma [25]. In addition, the scheme using predictions of TBV from SGM as selection criteria had a higher accuracy of GESC compared to the alternative schemes. However, this higher accuracy could be primarily attributed to a larger genetic variance that was capitalized on in the scheme using predictions of TBV as selection criteria.

When SGE were not used for selection, the use of the correctly specified model SGM increased RS when $r_{{u_{DS} }}$ was 0 or 0.5, but not when $r_{{u_{DS} }}$ was − 0.5. Based on our derivations (see “Appendix” section) and those of Chen et al*.* [5], selection criteria based on DGE when using CGM would have been: $u_{D}^{^{\prime}} = u_{D} - u_{S}$, which was in the wrong direction for SGE. In comparison, when using SGM with selection criteria based on DGE, selection was based on $u_{D}$, which could explain the higher RS in this case. Using the correctly specified model might also help to improve the accuracy of GESC when using SGM. However, these results could not explain the lower RS when using SGM with $r_{{u_{DS} }}$ =  − 0.5. One possible explanation is that, when using SGM, the ability of BLUP to separate DGE and SGE generally increases as $r_{{u_{DS} }}$ decreases, resulting in SGE to be less likely accounted for by selection on DGE. In contrast, because SGE was not included as an effect in CGM, the extent to which SGE is accounted for by selection on predictions of DGE may depend less on $r_{{u_{DS} }}$ for CGM than for SGM. Combining these two suggestions may explain the lower RS when using SGM when $r_{{u_{DS} }}$ is − 0.5.

With $\sigma_{{u_{S} }}^{2}$ = 0.001, i.e. SGE of low magnitude, inclusion of SGE in SGM did not improve RS, which might be due to the complexity of the model that makes it difficult to separate effects and hinders convergence. Consequently, the accuracy of GESC declined slightly compared to that of CGM [See Additional file 1: Table S2]. Default values for convergence criteria were set for the DMUAI procedure when the variance components were estimated and for the DMU5 procedure when the EBV were predicted [23]. However, for a number of replicates, the iterative solving methods for the SGM predictions of breeding values did not converge [See Additional file 1: Table S3]. Ødegård and Olesen [18] encountered convergence problems in 6 to 24% of replicates when using pedigree-based relationships with SGE to estimate variance components. Because a stochastic simulation using genomic-based models involves extensive computation, we did not investigate whether the iterative solving method converged for each replicate of the analyzed model. Yet, when overlooking computation costs, SGM allows the heritable variation from SGE to be capitalized on, thus improving RS, even at low magnitudes of SGE, for instance $\sigma_{{u_{S} }}^{2}$ = 0.001 relative to $\sigma_{{u_{D} }}^{2}$ = 1.

Muir [9] showed that RS increased when capitalizing SGE for selection, but in that study the rate of inbreeding was not considered. Khaw et al*.* [11] recorded significantly higher rates of inbreeding when using SGE for selection. However, neither of these studies [9, 11] investigated the impact of using SGE at the same rate of inbreeding or at the same rate of RS. Using OCS, the rate of inbreeding in our simulated pig breeding program was restricted to 1% per generation based on pedigree relationships. Note that this does not necessarily restrict the rate of true inbreeding to 1%. In Additional file 1: Table S5, RS was scaled to the rate of true inbreeding as $${\raise0.7ex\hbox{${RS}$} \!\mathord{\left/ {\vphantom {{RS} {\Delta F_{true} }}}\right.\kern-\nulldelimiterspace} \!\lower0.7ex\hbox{${\Delta F_{true} }$}}$$, which was RS per 1% increase in true inbreeding. RS across breeding schemes (Fig. 1) was similar to RS per 1% increase in true inbreeding (see Additional file 1: Table S5). Therefore, our results confirm that using SGE for selection improves RS without needing to sacrifice the rate of inbreeding.

We determined how the parameters in SGM were related to parameters in CGM (see “Appendix” section). The derivations were based on several assumptions. For example, parameters in SGM and CGM were assumed to be determined by differences between variance and covariance of phenotypes within a pen, as well as the average difference between the covariance of phenotypes within and between pens. This assumption is not completely correct, and there exist alternative derivations of how parameters in SGM relate to parameters in CGM. For instance, Bijma [25] showed that the residual variance differed between SGM and CGM as: $\sigma_{{e^{\prime}}}^{2} = \sigma_{{u_{S} }}^{2} - 2\sigma_{{u_{DS} }} + \sigma_{e}^{2}$; however, in our derivation, the two models had the same residual variance. The difference in pen variance between SGM and CGM in our derivation agreed with results of Bijma [25] and Bergsma et al*.* [2]: $\sigma_{{c_{p}^{^{\prime}} }}^{2} = \sigma_{{c_{p} }}^{2} + 2\sigma_{{u_{DS} }} + \sigma_{{u_{S} }}^{2} \left( {n - 2} \right)$. If the residual and pen variances from Bijma [25] and Bergsma et al*.* [2] were assumed, variance in DGE should not change when SGM or CGM is used. However, DGE, pen, and residual variances differed between SGM and CGM, as shown in Table 5. The difference in the parameters of SGM and CGM between our study and Bijma [25] was due to the assumptions of the two derivations. In our study, the genetic variances and covariances were assumed to be determined by the variation in relatedness between animals within pens. In contrast, Bijma [25] assumed the genetic variances and covariances were determined by the variation in relatedness of animals between pens.

Compared to pedigree-based relationships, using genomic-based relationships for predictions results in a substantial increase in the accuracy of DGE [20, 26–29] and TBV [30, 31]. The accuracy of TBV (i.e. accuracy of GESC in the scheme using predictions of TBV from SGM as selection criteria) was based on the correlation between predicted and true TBV. Genomic-based relationships provide better estimates of the actual genetic relationships between individuals [32], which increases not only the accuracy of DGE, but also that of SGE and TBV.

### Group composition

Differences in RS between random grouping and family grouping were smaller when genomic-based relationships were used than when pedigree-based relationships were used, which supports our hypothesis. This result could be explained by the capability of genomic-based relationships to exploit information from group members that are unrelated based on pedigree, which favors random grouping for predicting SGE. Nevertheless, RS was higher with family grouping, regardless of the relationships, statistical model, or selection criteria used in our study.

In the breeding scheme using predictions of TBV from SGM as selection criteria, a higher RS with family grouping could be explained by the higher accuracy of SGE. Yet, we observed a small difference in accuracy of DGE with random grouping. The higher accuracy of SGE with family grouping indicates that it is more important to obtain information on group members to predict SGE compared to information on animals from other groups. Thus, higher relatedness among group members likely increases the accuracy of SGE. Another reason for the increased accuracy could be due to the amplified effect of SGE when group members were composed of families. As an extreme example, for a group composed of $n$ clones, the phenotype of an individual would receive $n$* − *1 times its own SGE, which has a variance of $\left( {n - 1} \right)^{2} {*}\sigma_{{u_{S} }}^{2}$. In contrast, when a group is composed of $n$ unrelated individuals, the phenotype of an individual would be affected by SGE from group mates with a variance of $\left( {n - 1} \right){*}\sigma_{{u_{S} }}^{2}$. As the effect of SGE is much larger in groups composed of clones, it would be predicted more accurately.

Interestingly, in the two breeding schemes using predictions of DGE from SGM and CGM as selection criteria, RS was higher with family grouping than with random grouping. This could be explained by the effects of DGE and SGE being poorly separated when group members are composed of families. In other words, SGE might be partly confounded by DGE in this design, with SGE being partly accounted for in selection criteria when it is based on DGE predicted from SGM and CGM models.

The effects of group composition on prediction of genetic effects and variance estimation observed here are consistent with previous reports [17, 18]. Ødegård and Olesen [18] found that the accuracy of SGE and TBV was higher for groups composed of families, whereas the accuracy of DGE was lower for family groups compared to random groups. Compared to random groups, family groups had lower standard errors for estimates of SGE variance [17, 18], but high standard errors for estimates of DGE variance [18]. Our findings are also consistent with the results of Ødegård and Olesen [18] regarding the accuracy of TBV for random and family groups. However, these studies [17, 18] only considered SGM. When CGM was used, the accuracy of TBV was also higher with family grouping compared to random grouping. If $\left( {\sigma_{{u_{D} }}^{2} + \left( {n - 1} \right)\sigma_{{u_{DS} }} } \right) < 0$, random grouping could result in a negative accuracy of GESC [19, 25]; however, Ellen et al*.* [19] showed that the accuracy of GESC from CGM is always positive when group members are composed of families. In addition, our study shows that higher RS in groups composed of families could be obtained when predictions of DGE from SGM are used as the selection criteria, which had not been demonstrated to date.

## Conclusions

Our study compared statistical models that did or did not include predictions of SGE for the selection of a trait affected by social interactions in a breeding program for pigs, in which the rate of inbreeding was constrained. Statistical models that include SGE improved RS substantially, mainly because all the heritable variation from SGE could be exploited for selection. By using the correctly specified model, RS also increased slightly as a result of greater accuracy of predictions of DGE, depending on the genetic correlation between DGE and SGE. In addition, groups composed of four families were compared to randomly composed groups when using pedigree- or genomic-based relationships of animals. Family group composition resulted in higher RS, regardless of the models used. However, the relative difference in RS between the two group compositions declined when using genomic-based relationships. In summary, for production systems in which animals are commonly housed in groups and for traits that are affected by SGE, statistical models using SGE for selection substantially increase RS at a fixed rate of inbreeding, because the heritable variation from SGE is accounted for. The advantage of using family groups compared to random groups for RS reduces when using genomic-based relationships than pedigree-based relationships.

### Supplementary Information

**Additional file 1: Table S1.** Accuracies and bias of predicted genetic effects (mean ± standard error over 100 replicates) for the three breeding schemes assuming a social genetic variance ($\sigma_{{u_{S} }}^{2}$) of 0.01. The means and standard errors over 100 replicates are shown for the accuracies and bias of predicted genetic effects between the three breeding schemes: SGM_DGE used a social genetic model (SGM) with selection criteria based on direct genetic values (DGE); SGM_TBV used a SGM with selection criteria based on total breeding values (TBV); and CGM_DGE used the classical genetic model (CGM) with selection criteria based on DGE. The three breeding schemes were compared for traits with an SGE variance ($\sigma_{{u_{S} }}^{2}$) equal to 0.01. **Table S2.** Accuracies and bias of predicted genetic effects (mean ± standard error over 100 replicates) for the three breeding schemes assuming a social genetic variance ($\sigma_{{u_{S} }}^{2}$) of 0.001. The means and standard errors over 100 replicates are shown for the accuracies and bias of predicted genetic effects between the three breeding schemes: SGM_DGE used a social genetic model (SGM) with selection criteria based on direct genetic values (DGE); SGM_TBV used a SGM with selection criteria based on total breeding values (TBV); and CGM_DGE used the classical genetic model (CGM) with selection criteria based on DGE. The three breeding schemes were compared for traits with an SGE variance ($\sigma_{{u_{S} }}^{2}$) equal to 0.001. **Table S3.** Percentage of converged replicates in scenarios assuming a social genetic variance ($\sigma_{{u_{S} }}^{2}$) of 0.001 or 0.01, and a correlation ($r_{{u_{DS} }}$) between direct and social genetic effects at -0.5, 0 or 0.5. The table shows the convergence of the model for the estimation of variance components based on the average information (AI) restricted maximum likelihood (REML) estimation method (DMUAI module), and for solving BLUP equations based on the preconditioned conjugate gradient method (DMU5 module). **Table S4.** Accuracies of predicted genetic effects and bias in prediction (mean ± standard error over 100 replicates) when group members were composed at random *versus* composed of four families per group. The means and standard errors over 100 replicates are shown for the accuracies and bias of predicted genetic effects between family groups and random groups. These two designs were compared under three breeding schemes: SGM_DGE used a social genetic model (SGM) with selection criteria based on direct genetic values (DGE); SGM_TBV used a SGM with selection criteria based on total breeding values (TBV); and CGM_DGE used the classical genetic model (CGM) with selection criteria based on DGE. The trait was simulated with an SGE variance of 0.01 and a correlation between SGE and DGE of 0. **Table S5.** Response to selection per 1% of increase in true inbreeding (mean ± standard error over 100 replicates) in the three breeding schemes**.** The table shows response to selection per 1% of increase in true inbreeding for different breeding schemes using either pedigree-based or genomic-based relationship matrices. The three breeding schemes were compared for traits with an SGE variance ($\sigma_{{u_{S} }}^{2}$) equal to 0.01 and correlations ($r_{{u_{DS} }}$) between SGE and DGE of -0.5, 0, and 0.5. Group members were allocated at random or composed of families.

## Data Availability

The data that support the findings of this study are available from the corresponding author upon reasonable request.

## Appendix

### Genetic parameters in models with and without SGE

This Appendix presents the derivation that was used to show how the parameters for the model with SGE were related to those for the model without SGE. It was assumed that group members were composed at random, and litter effect was absent.

The model with SGE, called SGM, in the scalar notation was:

A1.1 $$y_{i} = \mu + u_{D,i} + \mathop \sum \limits_{{j \in p\backslash \left\{ i \right\}}}^{n} u_{S,j} + c_{p} + e_{i} ,$$

where $y_{i}$ was the phenotypic record of individual $i$ in pen $p$ that has n individuals; $\mu$ is the mean; $u_{D,i}$ is the direct breeding value (DGE) of individual $i$ using SGM; $\mathop \sum \nolimits_{{j \in g\backslash \left\{ i \right\}}}^{n} u_{S,j}$ is the sum of the social genetic effects (SGE) of pen mates $j$ in pen $p$, not including individual $i$; $c_{p}$ is the pen effect of pen $p$ using SGM; $e_{i}$ is the residual using SGM. Using SGM, variance of DGE is $\sigma_{{u_{D} }}^{2}$, variance of SGE is $\sigma_{{u_{S} }}^{2}$, covariance of DGE and SGE is $\sigma_{{u_{DS} }}$, variance of pen effect is $\sigma_{{c_{p} }}^{2}$, and residual variance is $\sigma_{e}^{2}$.

Consider two individuals $i_{1}$ and $i_{2}$ in pen $p_{1}$ and another individual $i_{3}$ in another pen $p_{2}$. Individuals $j$ and $j^{\prime}$ are pen mates of individual $i$. Phenotypic variance and covariance based on SGM would be:

$$\begin{aligned} Var\left( {y_{{i_{1} }} } \right) & = A_{{i_{1} i_{1} }} \sigma_{{u_{D} }}^{2} + 2\mathop \sum \limits_{{j \in p_{1} \backslash \left\{ {i_{1} } \right\}}} A_{{i_{1} j}} \sigma_{{u_{DS} }} + \mathop \sum \limits_{{j,j^{\prime} \in p_{1} \backslash \left\{ {i_{1} } \right\}}} A_{{jj^{\prime}}} \sigma_{{u_{S} }}^{2} + \sigma_{{c_{p} }}^{2} + \sigma_{e}^{2} , \\ Var\left( {y_{{i_{2} }} } \right) & = A_{{i_{2} i_{2} }} \sigma_{{u_{D} }}^{2} + 2\mathop \sum \limits_{{j \in p_{1} \backslash \left\{ {i_{2} } \right\}}} A_{{i_{2} j}} \sigma_{{u_{DS} }} + \mathop \sum \limits_{{j,j^{\prime} \in p_{1} \backslash \left\{ {i_{2} } \right\}}} A_{{jj^{\prime}}} \sigma_{{u_{S} }}^{2} + \sigma_{{c_{p} }}^{2} + \sigma_{e}^{2} , \\ Cov\left( {y_{{i_{1} }} , y_{{i_{2} }} } \right) & = A_{{i_{1} i_{2} }} \sigma_{{u_{D} }}^{2} + \left( {\mathop \sum \limits_{{j \in p_{1} \left\{ {i_{1} } \right\}}} A_{{i_{2} j}} + \mathop \sum \limits_{{j \in p_{1} \left\{ {i_{2} } \right\}}} A_{{i_{1} j}} } \right)\sigma_{{u_{DS} }} + \left( {\mathop \sum \limits_{{j \in p_{1} \backslash \left\{ {i_{1} } \right\}}} \mathop \sum \limits_{{j^{\prime} \in p_{1} \backslash \left\{ {i_{2} } \right\}}} A_{{jj^{\prime}}} } \right)\sigma_{{u_{S} }}^{2} + \sigma_{{c_{p} }}^{2} , \\ Cov\left( {y_{{i_{1} }} , y_{{i_{3} }} } \right) & = A_{{i_{1} i_{3} }} \sigma_{{u_{D} }}^{2} + \left( {\mathop \sum \limits_{{j \in p_{1} \left\{ {i_{1} } \right\}}} A_{{i_{3} j}} + \mathop \sum \limits_{{j \in p_{2} \left\{ {i_{3} } \right\}}} A_{{i_{1} j}} } \right)\sigma_{{u_{DS} }} + \left( {\mathop \sum \limits_{{j \in p_{1} \backslash \left\{ {i_{1} } \right\}}} \mathop \sum \limits_{{j^{\prime} \in p_{2} \backslash \left\{ {i_{3} } \right\}}} A_{{jj^{\prime}}} } \right)\sigma_{{u_{S} }}^{2} . \\ \end{aligned}$$

where $A$ is the relationship coefficient between two animals.

The model without SGE, called CGM, in scalar notation is:

A1.2 $$y_{i} = \mu + u_{D,i}^{^{\prime}} + c_{p}^{^{\prime}} + e_{i}^{^{\prime}} ,$$

where $u_{D,i}^{^{\prime}}$ is the direct breeding value (DGE) of individual $i$ using CGM; $c_{p}^{^{\prime}}$ is the pen effect of pen $p$ using CGM; and $e_{i}$ is the residual $e_{i}^{^{\prime}}$. Variance of DGE is $\sigma_{{u_{D}^{^{\prime}} }}^{2}$, variance of pen effect is $\sigma_{{c_{p}^{^{\prime}} }}^{2}$, and residual variance is $\sigma_{{e^{\prime}}}^{2}$.

Phenotypic variances and covariances based on CGM would be:

$$\begin{aligned} Var\left( {y_{{i_{1} }} } \right) &= A_{{i_{1} i_{1} }} \sigma_{{u_{D}^{^{\prime}} }}^{2} + \sigma_{{c_{p}^{^{\prime}} }}^{2} + \sigma_{{e^{\prime}}}^{2} , \hfill \\ Var\left( {y_{{i_{2} }} } \right) &= A_{{i_{2} i_{2} }} \sigma_{{u_{D}^{^{\prime}} }}^{2} + \sigma_{{c_{p}^{^{\prime}} }}^{2} + \sigma_{{e^{\prime}}}^{2} , \hfill \\ Cov\left( {y_{{i_{1} }} ,y_{{i_{2} }} } \right) &= A_{{i_{1} i_{2} }} \sigma_{{u_{D}^{^{\prime}} }}^{2} + \sigma_{{c_{p}^{^{\prime}} }}^{2} , \hfill \\ Cov\left( {y_{{i_{1} }} ,y_{{i_{3} }} } \right) &= A_{{i_{1} i_{3} }} \sigma_{{u_{D}^{^{\prime}} }}^{2} . \hfill \\ \end{aligned}$$

We see that information about the residual variance is in the differences between $Var\left( {y_{{i_{1} }} } \right)$ and $Cov\left( {y_{{i_{1} }} ,y_{{i_{2} }} } \right)$, and information about the group variance is in the difference between $Cov\left( {y_{{i_{1} }} ,y_{{i_{2} }} } \right)$, i.e. covariance between pen members, and $Cov\left( {y_{{i_{1} }} ,y_{{i_{3} }} } \right)$, i.e. covariance between individuals that are not pen members. Thus, we study such differences.

First, in SGM, after some algebra the difference between variances and covariance between pen members equals:

A1.3 $$\frac{{Var\left( {y_{{i_{1} }} } \right) + Var\left( {y_{{i_{2} }} } \right)}}{2} - Cov\left( {y_{{i_{1} }} ,y_{{i_{2} }} } \right) = \left( {\frac{{A_{{i_{1} i_{1} }} + A_{{i_{2} i_{2} }} }}{2} - A_{{i_{1} i_{2} }} } \right)\left( {\sigma_{{u_{D} }}^{2} - 2\sigma_{{u_{DS} }} + \sigma_{{u_{S} }}^{2} } \right) + \sigma_{e}^{2} .$$

In CGM, this becomes:

A1.4 $$\frac{{Var\left( {y_{{i_{1} }} } \right) + Var\left( {y_{{i_{2} }} } \right)}}{2} - Cov\left( {y_{{i_{1} }} ,y_{{i_{2} }} } \right) = \left( {\frac{{A_{{i_{1} i_{1} }} + A_{{i_{2} i_{2} }} }}{2} - A_{{i_{1} i_{2} }} } \right)\sigma_{{u_{D}^{^{\prime}} }}^{2} + \sigma_{{e^{\prime}}}^{2} .$$

Due to the random composition of groups, then there is variation in relatedness among pairs of group mates, and therefore the term $\frac{{A_{{i_{1} i_{1} }} + A_{{i_{2} i_{2} }} }}{2} - A_{{i_{1} i_{2} }}$ differs between pairs of individuals in a pen. Comparing these two equations (A1.3) and (A1.4) and noting that they hold for any pair of individuals in the same pen, suggests that in CGM, an expression for the direct genetic variance is a function of parameters in SGM: $\sigma_{{u_{D}^{^{\prime}} }}^{2} = \sigma_{{u_{D} }}^{2} - 2\sigma_{{u_{DS} }} + \sigma_{{u_{S} }}^{2}$, whereas the residual variance in the two models is the same.

Second, in SGM, by averaging over all pairs of individuals in the same pen and over all pens, the phenotypic covariance between $i_{1}$ and $i_{2}$ is:

$$\begin{aligned} \overline{{Cov\left( {y_{{i_{1} }} , y_{{i_{2} }} } \right)}} & = \overline{{A_{0,w} }} \sigma_{{u_{D} }}^{2} + 2\left( {\overline{dA} + \overline{{A_{0,w} }} \left( {n - 2} \right)} \right)\sigma_{{u_{DS} }} \\ & \quad + \left( {\overline{dA} \left( {n - 2} \right) + \overline{{A_{0,w} }} \left( {\left( {n - 1} \right)^{2} - \left( {n - 2} \right)} \right)} \right)\sigma_{{u_{S} }}^{2} + \sigma_{{c_{p} }}^{2} . \\ \end{aligned}$$

where $\overline{dA}$ is the average self-relationship and $\overline{{A_{0,w} }}$ is the average relationship between pen mates, and averaging over all pairs of individuals in different pens and all pairs of different pens,

$$\overline{{Cov\left( {y_{{i_{1} }} , y_{{i_{3} }} } \right)}} = \overline{{A_{0,b} }} \sigma_{{u_{D} }}^{2} + 2\overline{{A_{0,b} }} \left( {n - 1} \right)\sigma_{{u_{DS} }} + \overline{{A_{0,b} }} \left( {n - 1} \right)^{2} \sigma_{{u_{S} }}^{2} ,$$

where $\overline{{A_{0,b} }}$ is the average relationship between individuals in different pens. Assuming that relationships within pens are the same as relationships between pens, $\overline{{A_{0,b} }} = \overline{{A_{0,w} }} = \overline{{A_{0} }}$, as is the case when animals are assigned randomly to pens, then the difference

A1.5 $$\overline{{Cov\left( {y_{{i_{1} }} , y_{{i_{2} }} } \right)}} - \overline{{Cov\left( {y_{{i_{1} }} , y_{{i_{3} }} } \right)}} = 2\left( {\overline{dA} - \overline{{A_{0} }} } \right)\sigma_{{u_{DS} }} + \left( {n - 2} \right)\left( {\overline{dA} - \overline{{A_{0} }} } \right)\sigma_{{u_{S} }}^{2} + \sigma_{{c_{p} }}^{2} ,$$

The expression for CGM is:

A1.6 $$\overline{{Cov\left( {y_{{i_{1} }} , y_{{i_{2} }} } \right)}} - \overline{{Cov\left( {y_{{i_{1} }} , y_{{i_{3} }} } \right)}} = \sigma_{{c_{p}^{^{\prime}} }}^{2} .$$

The comparison between these two equations (A1.5) and (A1.6) suggests that in CGM, an expression for the random pen variance as a function of parameters in the social genetic model is:

$$\sigma_{{c_{p}^{^{\prime}} }}^{2} = 2\left( {\overline{dA} - \overline{{A_{0} }} } \right)\sigma_{{u_{DS} }} + \left( {n - 2} \right)\left( {\overline{dA} - \overline{{A_{0} }} } \right)\sigma_{{u_{S} }}^{2} + \sigma_{{c_{p} }}^{2} .$$

A simplification is obtained when relationships and self-relationships are weak, in which case $\overline{dA} - \overline{{A_{0} }} \approx 1,$ and this gives the expression:

$$\sigma_{{c_{p}^{^{\prime}} }}^{2} = 2\sigma_{{u_{DS} }} + \sigma_{{u_{S} }}^{2} \left( {n - 2} \right) + \sigma_{{c_{p} }}^{2} .$$

Note that SGM and CGM are not completely determined by differences between variance and covariance within pen, and averages of differences between covariances within pens and covariances between pens. Therefore, alternative equations for relating parameters in SGM to parameters in CGM can be derived instead.
