# Supplementary material for: Statistical model and testing designs to increase response to selection with constrained inbreeding in genomic breeding programs for pigs affected by social genetic effects
Source: Genet Sel Evol. 2021 Jan 4;53:1. doi: 10.1186/s12711-020-00598-8 (PMC7784391; doi:10.1186/s12711-020-00598-8)
Supplement: Supplementary file 1 — Additional file 1: Table S1. Accuracies and bias of predicted genetic effects (mean ± standard error over 100 replicates) for the three breeding schemes assuming a social genetic variance ($\sigma_{{u_{S} }}^{2}$σuS2) of 0.01. The means and standard errors over 100 replicates are shown for the accuracies and bias of predicted genetic effects between the three breeding schemes: SGM_DGE used a social genetic model (SGM) with selection criteria based on direct genetic values (DGE); SGM_TBV used a SGM with selection criteria based on total breeding values (TBV); and CGM_DGE used the classical genetic model (CGM) with selection criteria based on DGE. The three breeding schemes were compared for traits with an SGE variance ($\sigma_{{u_{S} }}^{2}$σuS2) equal to 0.01. Table S2. Accuracies and bias of predicted genetic effects (mean ± standard error over 100 replicates) for the three breeding schemes assuming a social genetic variance ($\sigma_{{u_{S} }}^{2}$σuS2) of 0.001. The means and standard errors over 100 replicates are shown for the accuracies and bias of predicted genetic effects between the three breeding schemes: SGM_DGE used a social genetic model (SGM) with selection criteria based on direct genetic values (DGE); SGM_TBV used a SGM with select [file 12711_2020_598_MOESM1_ESM.docx]

**Table S1** **Accuracies and bias of predicted genetic effects (mean ± standard error over 100 replicates) for the three breeding schemes assuming social genetic variance (**$\boldsymbol{\sigma}_{\boldsymbol{u}_{\boldsymbol{S}}}^{\mathbf{2}}$**) of 0.01**

|  | **A** |  |  | **G** |  |  |
| --- | --- | --- | --- | --- | --- | --- |
| $r_{u_{DS}}$ | SGM_DGE | SGM_TBV | CGM_DGE | SGM_DGE | SGM_TBV | CGM_DGE |
| Accuracy of GESC | | |  |  |  |  |
| -0.5 | 0.201±0.007 | 0.318±0.008 | 0.204±0.009 | 0.244±0.006 | 0.437±0.007 | 0.269±0.007 |
| 0 | 0.347±0.008 | 0.430±0.006 | 0.341±0.007 | 0.449±0.005 | 0.556±0.005 | 0.436±0.005 |
| 0.5 | 0.481±0.005 | 0.504±0.005 | 0.457±0.005 | 0.614±0.004 | 0.656±0.004 | 0.596±0.004 |
| Accuracy of DGE | |  |  |  |  |  |
| -0.5 | 0.582±0.004 | 0.623±0.004 | 0.588±0.004 | 0.77±0.002 | 0.811±0.002 | 0.774±0.002 |
| 0 | 0.583±0.004 | 0.604±0.004 | 0.580±0.004 | 0.768±0.002 | 0.792±0.002 | 0.765±0.002 |
| 0.5 | 0.580±0.004 | 0.586±0.004 | 0.577±0.004 | 0.765±0.002 | 0.777±0.002 | 0.764±0.002 |
| Bias of GESC | |  |  |  |  |  |
| -0.5 | 0.39±0.01 | 1.00±0.04 | 0.39±0.02 | 0.38±0.01 | 0.98±0.03 | 0.41±0.01 |
| 0 | 0.92±0.02 | 1.01±0.03 | 0.94±0.02 | 0.94±0.01 | 0.99±0.02 | 0.93±0.01 |
| 0.5 | 1.54±0.02 | 1.05±0.02 | 1.50±0.02 | 1.52±0.01 | 1.00±0.02 | 1.51±0.01 |
| Bias of DGE | |  |  |  |  |  |
| -0.5 | 0.99±0.01 | 0.99±0.01 | 0.97±0.01 | 1.00±0.01 | 1.01±0.01 | 0.97±0.01 |
| 0 | 0.98±0.01 | 0.99±0.01 | 1.01±0.01 | 0.99±0.01 | 1.00±0.01 | 1.00±0.01 |
| 0.5 | 0.99±0.01 | 1.00±0.01 | 1.02±0.01 | 0.99±0.01 | 1.00±0.01 | 1.02±0.01 |

Note: SGM_DGE used a social genetic model (SGM) with selection criteria based on direct genetic values (DGE); SGM_TBV used a SGM with selection criteria based on total breeding values (TBV); and CGM_DGE used the classical genetic model (CGM) with selection criteria based on DGE. Breeding schemes used either pedigree- (**A**) or genomic-based (**G**) relationships to predict genetic effects. The three breeding schemes was compared using different assumptions of the trait simulated with correlation ($r_{u_{DS}}$) between SGE and DGE at -0.5, 0, and 0.5. Group members were allocated at random. Accuracy and bias of predicted genetic effects were computed based on animals at generation t = 4.

Accuracy of predicted genetic effects of selection criteria (GESC) was calculated as the correlation between GESC and true TBV. Bias of GESC was the regression coefficients of true values of TBV on predicted values of GESC.

**Table S2 Accuracies and bias of predicted genetic effects (mean ± standard error over 100 replicates) for the three breeding schemes assuming social genetic variance (**$\boldsymbol{\sigma}_{\boldsymbol{u}_{\boldsymbol{S}}}^{\mathbf{2}}$**) of 0.001**

|  | **A** |  |  | **G** |  |  |
| --- | --- | --- | --- | --- | --- | --- |
| $r_{u_{DS}}$ | SGM_DGE | SGM_TBV | CGM_DGE | SGM_DGE | SGM_TBV | CGM_DGE |
| Accuracy of GESC | | |  |  |  |  |
| -0.5 | 0.537±0.004 | 0.534±0.005 | 0.543±0.005 | 0.700±0.003 | 0.696±0.003 | 0.701±0.003 |
| 0 | 0.532±0.004 | 0.542±0.004 | 0.539±0.005 | 0.705±0.003 | 0.699±0.003 | 0.707±0.003 |
| 0.5 | 0.559±0.004 | 0.552±0.005 | 0.557±0.004 | 0.737±0.003 | 0.725±0.003 | 0.733±0.002 |
| Accuracy of DGE | |  |  |  |  |  |
| -0.5 | 0.579±0.004 | 0.588±0.004 | 0.587±0.004 | 0.769±0.002 | 0.774±0.002 | 0.770±0.002 |
| 0 | 0.576±0.003 | 0.593±0.003 | 0.583±0.004 | 0.769±0.002 | 0.769±0.002 | 0.770±0.002 |
| 0.5 | 0.583±0.004 | 0.580±0.004 | 0.580±0.004 | 0.771±0.002 | 0.770±0.002 | 0.771±0.002 |
| Bias of GESC | |  |  |  |  |  |
| -0.5 | 0.82±0.01 | 1.06±0.03 | 0.82±0.01 | 0.84±0.02 | 0.99±0.02 | 0.81±0.01 |
| 0 | 0.99±0.01 | 0.96±0.02 | 0.97±0.01 | 0.99±0.01 | 0.99±0.02 | 0.98±0.01 |
| 0.5 | 1.20±0.01 | 0.97±0.02 | 1.19±0.01 | 1.17±0.01 | 1.01±0.02 | 1.17±0.01 |
| Bias of DGE | |  |  |  |  |  |
| -0.5 | 1.00±0.01 | 1.01±0.01 | 0.99±0.01 | 1.03±0.03 | 1.00±0.01 | 0.99±0.01 |
| 0 | 1.01±0.01 | 0.98±0.01 | 0.98±0.01 | 1.00±0.01 | 0.99±0.01 | 0.99±0.01 |
| 0.5 | 1.01±0.01 | 0.98±0.01 | 1.02±0.01 | 1.01±0.01 | 1.00±0.01 | 1.01±0.01 |

Note: SGM_DGE used a social genetic model (SGM) with selection criteria based on direct genetic values (DGE); SGM_TBV used a SGM with selection criteria based on total breeding values (TBV); and CGM_DGE used the classical genetic model (CGM) with selection criteria based on DGE. Breeding schemes used either pedigree- (**A**) or genomic-based (**G**) relationships to predict genetic effects. The three breeding schemes was compared using different assumptions of the trait simulated with correlation ($r_{u_{DS}}$) between SGE and DGE at -0.5, 0, and 0.5. Group members were allocated at random. Accuracy and bias of predicted genetic effects were computed based on animals at generation t = 4.

Accuracy of predicted genetic effects of selection criteria (GESC) was calculated as the correlation between GESC and true TBV. Bias of GESC was the regression coefficients of true values of TBV on predicted values of GESC.

**Table S3 Percentage of replicates converged in scenarios assuming social genetic variance (**$\boldsymbol{\sigma}_{\boldsymbol{u}_{\boldsymbol{S}}}^{\boldsymbol{2}}$**) of 0.001 or 0.01, and correlation (**$\boldsymbol{r}_{\boldsymbol{u}_{\boldsymbol{DS}}}$**) between direct and social genetic effects at -0.5, 0 or 0.5.**

|  |  | $\sigma_{u_{S}}^{2}=0.001$ |  |  | $\sigma_{u_{S}}^{2}=0.01$ |  |  |
| --- | --- | --- | --- | --- | --- | --- | --- |
| Module | *t* | $r_{u_{DS}}=-0.5$ | $r_{u_{DS}}=0$ | $r_{u_{DS}}=0.5$ | $r_{u_{DS}}=-0.5$ | $r_{u_{DS}}=0$ | $r_{u_{DS}}=0.5$ |
| DMUAI | 3 | 97.5 | 99.1 | 97.4 | 97.7 | 100 | 98.2 |
| DMU5 | 3 | 59.5 | 63.2 | 74.4 | 98.1 | 98.7 | 98.2 |
| DMU5 | 4 | 56.3 | 61.5 | 70.1 | 98.1 | 98.7 | 97.6 |
| DMU5 | 5 | 55.1 | 58.1 | 63.8 | 97.7 | 98.7 | 97.6 |
| DMU5 | 6 | 53.0 | 55.3 | 61.0 | 97.1 | 98.7 | 96.7 |
| DMU5 | 7 | 44.4 | 44.7 | 53.1 | 95.5 | 96.7 | 94.5 |
| DMU5 | 8 | 32.3 | 32.7 | 42.5 | 91.9 | 93.1 | 88.1 |
| DMU5 | 9 | 25.5 | 23.0 | 33.8 | 85.7 | 88.2 | 79.7 |
| DMU5 | 10 | 19.5 | 20.4 | 32.0 | 75.0 | 81.2 | 70.2 |

Note: The convergence was for the estimation of variance components based on the average information (AI) restricted maximum likelihood (REML) estimation method (DMUAI module), and for solving BLUP equations based on the preconditioned conjugate gradient method (DMU5 module) at generation *t*. The scenarios used random group composition.

**Table S4 Accuracies and bias of predicted genetic effects (mean ± standard error over 100 replicates) when group members were composed at random *versus* composed of four families per group**

|  | **A** |  |  |  | **G** |  |  |  |
| --- | --- | --- | --- | --- | --- | --- | --- | --- |
| Scheme | Fam_T | Fam_E | Ran_T | Ran_E | Fam_T | Fam_E | Ran_T | Ran_E |
| Accuracy of GESC | |  |  |  |  |  |  |  |
| SGM_DGE | 0.369  ±0.006 | 0.369  ±0.006 | 0.358  ±0.007 | 0.347  ±0.008 | 0.463  ±0.005 | 0.459  ±0.005 | 0.453  ±0.005 | 0.449  ±0.005 |
| SGM_TBV | 0.495  ±0.005 | 0.478  ±0.005 | 0.436  ±0.006 | 0.43  ±0.006 | 0.617  ±0.004 | 0.610  ±0.004 | 0.566  ±0.005 | 0.556  ±0.005 |
| CGM_DGE | n/a | 0.377  ±0.007 | n/a | 0.341  ±0.007 | n/a | 0.463  ±0.004 | n/a | 0.436  ±0.005 |
| Accuracy of DGE | | | | | | | | |
| SGM_DGE | 0.570  ±0.004 | 0.574  ±0.004 | 0.586  ±0.004 | 0.583  ±0.004 | 0.767  ±0.002 | 0.763  ±0.002 | 0.772  ±0.002 | 0.768  ±0.002 |
| SGM_TBV | 0.611  ±0.004 | 0.603  ±0.004 | 0.607  ±0.004 | 0.604  ±0.004 | 0.790  ±0.002 | 0.794  ±0.002 | 0.789  ±0.002 | 0.792  ±0.002 |
| CGM_DGE | n/a | 0.577  ±0.004 | n/a | 0.580  ±0.004 | n/a | 0.765  ±0.002 | n/a | 0.765  ±0.002 |
| Accuracy of SGE | | | | | | | | |
| SGM_TBV | 0.444  ±0.006 | 0.430  ±0.007 | 0.366  ±0.009 | 0.365  ±0.009 | 0.563  ±0.006 | 0.549  ±0.005 | 0.484  ±0.007 | 0.451  ±0.010 |
| Bias of GESC | |  |  |  |  |  |  |  |
| SGM_DGE | 1.01  ±0.02 | 0.99  ±0.02 | 0.95  ±0.02 | 0.92  ±0.02 | 0.97  ±0.01 | 0.97  ±0.01 | 0.94  ±0.01 | 0.94  ±0.01 |
| SGM_TBV | 1.00  ±0.01 | 1.01  ±0.02 | 0.97  ±0.01 | 1.01  ±0.03 | 0.98  ±0.01 | 1.00  ±0.01 | 0.98  ±0.01 | 0.99  ±0.02 |
| CGM_DGE | n/a | 1.02  ±0.02 | n/a | 0.94  ±0.02 | n/a | 0.97  ±0.01 | n/a | 0.93  ±0.01 |
| Bias of DGE | | | | | | | | |
| SGM_DGE | 0.99  ±0.01 | 0.99  ±0.01 | 0.99  ±0.01 | 0.98  ±0.01 | 0.99  ±0.00 | 0.99  ±0.01 | 0.99  ±0.00 | 0.99  ±0.01 |
| SGM_TBV | 0.99  ±0.00 | 0.98  ±0.01 | 0.99  ±0.00 | 0.99  ±0.01 | 0.98  ±0.00 | 1.00  ±0.01 | 0.99  ±0.00 | 1.00  ±0.01 |
| CGM_DGE | n/a | 1.01  ±0.01 | n/a | 1.01  ±0.01 | n/a | 0.98  ±0.01 | n/a | 1.00  ±0.01 |
| Bias of SGE | | | | | | | | |
| SGM_TBV | 1.00  ±0.01 | 0.97  ±0.02 | 0.97  ±0.02 | 1.05  ±0.05 | 1.00  ±0.01 | 1.01  ±0.02 | 0.98  ±0.02 | 1.07  ±0.09 |

Note: Fam_T and Fam_E are the scenario using family (Fam_) group composition and true (T) or estimated (E) variance components for prediction. Ran_T and Ran_E are the scenario using random (Ran_) group composition and T or E variance components.

These two designs were compared under three breeding schemes: SGM_DGE used a social genetic model (SGM) with selection criteria based on direct genetic values (DGE); SGM_TBV used a SGM with selection criteria based on total breeding values (TBV); and CGM_DGE used the classical genetic model (CGM) with selection criteria based on DGE. Breeding schemes used either pedigree- (**A**) or genomic-based (**G**) relationships to predict genetic effects. The trait was simulated with SGE variance of 0.01 and correlation between SGE and DGE of 0.

Accuracy of SGE and bias of SGE are not available (n/a) for model CGM.

**Table S5** **Response to selection per 1% of the increase in true inbreeding (mean ± standard error over 100 replicates) in the three breeding schemes**

| Relat. | Group composition | VC | $r_{u_{DS}}$ | SGM_DGE | SGM_TBV | CGM_DGE |
| --- | --- | --- | --- | --- | --- | --- |
| Comparing CGM and SGM | | |  |  |  |  |
| **A** | RAN | Estimated | -0.5 | 0.285±0.005 | 0.430±0.007 | 0.303±0.005 |
| **A** | RAN | Estimated | 0 | 0.715±0.007 | 0.829±0.008 | 0.696±0.006 |
| **A** | RAN | Estimated | 0.5 | 1.146±0.008 | 1.188±0.008 | 1.118±0.007 |
| **G** | RAN | Estimated | -0.5 | 0.333±0.005 | 0.515±0.007 | 0.343±0.004 |
| **G** | RAN | Estimated | 0 | 0.818±0.006 | 0.959±0.008 | 0.784±0.006 |
| **G** | RAN | Estimated | 0.5 | 1.302±0.008 | 1.360±0.009 | 1.271±0.009 |
| Comparing group composition | | |  |  |  |  |
| **A** | RAN | Estimated | 0 | 0.715±0.007 | 0.829±0.008 | 0.696±0.006 |
| **A** | FAM | Estimated | 0 | 0.734±0.006 | 0.920±0.007 | 0.748±0.007 |
| **A** | RAN | True | 0 | 0.712±0.007 | 0.856±0.006 | n/a |
| **A** | FAM | True | 0 | 0.748±0.006 | 0.916±0.007 | n/a |
| **G** | RAN | Estimated | 0 | 0.818±0.006 | 0.959±0.008 | 0.784±0.006 |
| **G** | FAM | Estimated | 0 | 0.833±0.006 | 1.038±0.007 | 0.829±0.007 |
| **G** | RAN | True | 0 | 0.814±0.007 | 0.978±0.007 | n/a |
| **G** | FAM | True | 0 | 0.835±0.005 | 1.034±0.007 | n/a |

Note: SGM_DGE used a social genetic model (SGM) with selection criteria based on direct genetic values (DGE); SGM_TBV used a SGM with selection criteria based on total breeding values (TBV); and CGM_DGE used the classical genetic model (CGM) with selection criteria based on DGE.

Group members were composed at random (RAN) or composed of four families per group (FAM). The trait was simulated with SGE variance of 0.01 and correlation ($r_{u_{DS}}$) between SGE and DGE at -0.5, 0, and 0.5.

Relat. is the relationship matrix used to predict genetic effects, either pedigree- (**A**) or genomic-based (**G**) relationships. VC is the variance component used, either true values or estimated values. Scenarios with the use of CGM and true variance components are not available (n/a) because the true variance components are unknown for CGM.
